# Women in space: A review of known physiological adaptations and health perspectives

**DOI:** 10.1113/EP091527

**Published:** 2024-11-02

**Authors:** Millie Hughes‐Fulford, Danielle J. Carroll, Heather C. M. Allaway, Bonnie J. Dunbar, Aenor J. Sawyer

**Affiliations:** ^1^ UC Space Health University of California San Francisco (UCSF) San Francisco California USA; ^2^ Department of Medicine UCSF San Francisco California USA; ^3^ Department of Surgery UCSF San Francisco California USA; ^4^ Department of Bioastronautics University of Colorado Boulder Boulder Colorado USA; ^5^ Department of Kinesiology Texas A&M University College Station Texas USA; ^6^ School of Kinesiology Louisiana State University Baton Rouge Louisiana USA; ^7^ Department of Aerospace Engineering Texas A&M University College Station Texas USA; ^8^ Texas A&M Engineering Experiment Station Texas A&M University College Station Texas USA; ^9^ Department of Orthopaedic Surgery UCSF San Francisco California USA

**Keywords:** exploration, gender, microgravity, physiology, women

## Abstract

Exposure to the spaceflight environment causes adaptations in most human physiological systems, many of which are thought to affect women differently from men. Since only 11.5% of astronauts worldwide have been female, these issues are largely understudied. The physiological nuances affecting the female body in the spaceflight environment remain inadequately defined since the last thorough published review on the subject. A PubMed literature search yielded over 2200 publications. Using NASA's 2014 review series ‘The effects of sex and gender on adaptation to space’ as a benchmark, we identified substantive advancements and persistent knowledge gaps in need of further study from the nearly 600 related articles that have been published since the initial review. This review highlights the most critical issues to mitigate medical risk and promote the success of missions to the Moon and Mars. Salient sex‐linked differences observed terrestrially should be studied during upcoming missions, including increased levels of inflammatory markers, coagulation factors and leptin levels following sleep deprivation; correlation between body mass and the severity of spaceflight‐associated neuro‐ocular syndrome; increased incidence of orthostatic intolerance; increased severity of muscle atrophy and bone loss; differences in the incidence of urinary tract infections; and susceptibility to specific cancers after exposure to ionizing radiation. To optimize health and well‐being among all astronauts, it is imperative to prioritize research that considers the physiological nuances of the female body. A more robust understanding of female physiology in the spaceflight environment will support crew readiness for Artemis missions and beyond.

## Abstract (highlights)

- **What is the topic of this review?**
  The limited expansion of our understanding of female astronaut physiology since 2014.
- **What advances does it highlight?**
  With female astronauts logging extensive flight time, over 7317 days and climbing in space since Valentina Tereskova's flight in 1963, it is imperative that female‐focused physiology research is expanded to fully understand the impact of biological sex on known adaptations to the spaceflight environment. This knowledge will enable the optimization of countermeasure development and maximize operational success for future missions to the Moon and Mars.

## 1 INTRODUCTION

From the earliest days of human flight, aviation has involved a continuous and dynamic analysis of risk and response in the context of aircraft capabilities, changing environmental factors and mission requirements. Over the course of the last seven decades of human space exploration, missions beyond the confines of Earth's atmosphere have differed little in this regard, save for a surge in the number and magnitude of threats with increasing mission duration and distance from Earth. The National Aeronautics and Space Administration (NASA), in collaboration with industry and academic partners, has made great strides in quantifying and mitigating risks to astronauts during missions in low Earth orbit or cislunar space, from which return to Earth is possible within a matter of days. Mars has been designated a long‐range target for extensive exploration on deep space missions – a goal that necessitates a critical reevaluation of preflight preparation and inflight health maintenance to facilitate effective mission completion.

A mission to Mars carries with it many implications for crews, both en route and on the planetary surface. In the context of the current Mars exploration design reference architecture, total mission duration may exceed 900 days in order to achieve a global minimum‐energy solution for a given launch opportunity: with existing propulsion capabilities, minimum transit time between planets is approximately 210 days, and an average stay of 500 days or more on the Martian surface will be required to permit entrance into an optimal return trajectory (Drake, 2009). The distance between Earth and Mars not only prohibits immediate evacuation or resupply but also exceeds our current capability for synchronous communication. Therefore, real‐time, acute medical assistance from any external source will not be available. With this in mind, NASA has ample motivation to optimize astronaut health prior to and during missions, in order to minimize the likelihood of medical conditions arising that might require emergent, resource‐intensive medical intervention.

NASA's human health risk mitigation plans are informed by prior flight data and risk modeling, as well as by Earth‐based healthcare and research. In the study of terrestrial health, there exist numerous examples of sex differences in health risks and treatment responses. In space, as on Earth, there is a marked paucity of sex‐specific medical knowledge, but its importance is expanding rapidly as mission crews continue to diversify.

At present, gender differences are understudied, perhaps in part because, as of March 2023, only 72 of the 622 (11.5%) space explorers, with a minimum of 24 h of flight time, have been women (Uri, 2023). A comprehensive review of women's health in space was published in 2014 (Mark et al., 2014), with few publications and limited tangible advancements occurring since then. We set out to write an updated review on the advancement of knowledge surrounding female astronaut health since the 2014 comprehensive review. We searched the NASA Technical Reports Server, which included research reports as well as onboard investigations and reported incidents. Additionally, we searched PubMed for literature on specific topics from the years 1963–2022 (including articles in press). Spaceflight, microgravity, head down bed rest, astronaut, hindlimb unloading, and galactic cosmic radiation (GCR) were terms used in conjunction with terms specific to each health/organ system section (i.e., ocular, cardiovascular (CV), skeletal muscle, bone, reproduction). Our PubMed literature searches yielded over 2200 articles written over the last 57 years. We paid special attention to the nearly 600 articles published since NASA's 2014 review entitled, ‘The effects of sex and gender on adaptation to space’. All articles were evaluated for any description of sex differences, for inclusion of female subjects, and for projects on female health specifically. Using the 2014 review as a benchmark, we identified substantive advancements as well as persistent knowledge gaps in clear need of further study.

**FIGURE 1:**
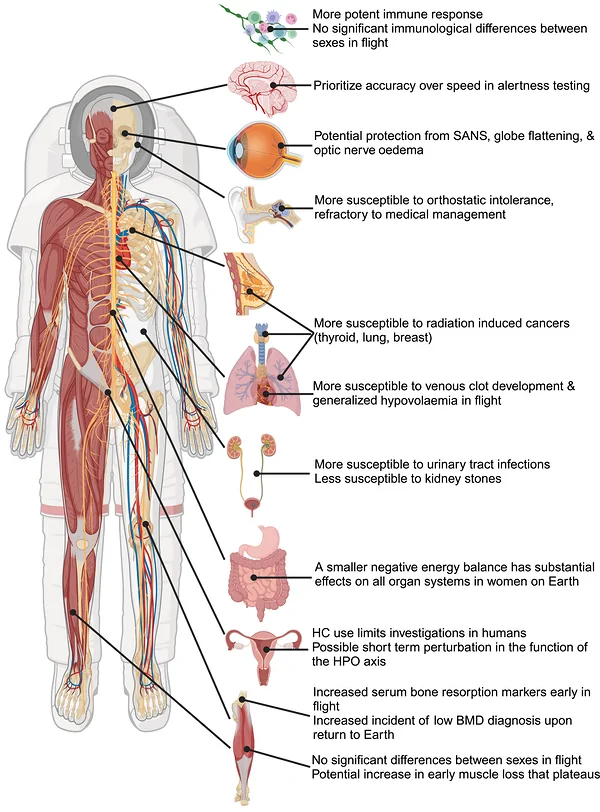
Known effects of the spaceflight environment on physiological systems in women. BMD, bone mineral density; HC, hormonal contraception; HPO, hypothalamic–pituitary–ovarian. (created using BioRender.com/r09k419)

Key areas in which critical, clinically significant sex‐based differences exist and pose challenges for health maintenance in space include ocular, CV, and musculoskeletal elements (Figure 1). Many cross‐cutting themes exist, most notably involving the effects of radiation exposure and the need for research focused on female adaptations to the space environment. Unlike terrestrial radiation, ionizing radiation (IR) in the form of GCR has dramatic and deleterious effects on tissues throughout the body, both during and after exposures in space. With exploratory missions to Mars on the near horizon, identifying opportunities to minimize radiation exposure will be critical for the preservation of astronaut health. Though preliminary investigations in terrestrial human studies and rodent models provide some insight, extensive longitudinal, sex‐specific investigations will be critical to fully understanding the physiological impacts of the spaceflight environment on all astronauts.

## 2 OCULAR

Ocular health of astronauts has garnered much attention in recent years (space environmental impacts summarized in Table 1). Observed effects up to now have included globe flattening, choroidal and retinal folds, hyperopic shifts in refractive error as great as +1.75 diopters, optic disc oedema of varying Frisen grades, and nerve fiber layer infarcts (i.e., ‘cotton wool spots’) (Lee et al., 2018). This constellation of findings is known as spaceflight‐associated neuro‐ocular syndrome (SANS) and has been linked to structural changes in the orbit as well as the globe itself. Almost uniformly, these effects become more pronounced as mission duration lengthens. The pathophysiology of this disease process appears to be multifactorial in nature, with likely combined effects from cephalad interstitial fluid shifts, venous congestion and stasis seen in the head and neck, cerebral lymphatic congestion, and elevated cerebrospinal fluid (CSF) pressure in the intracranial subarachnoid space (Lee et al., 2020), not a buildup of CSF pressure in the brain alone, as the development of optic disc oedema appears to be uncoupled from intracranial changes (Marshall‐Goebel et al., 2021). Recently, a positive correlation between body mass and severity of SANS was identified as indicated by changes in refractive error (*t *= 2.6, *P *= 0.012), suggesting that, while no direct relationship to sex was noted, female sex might indirectly play a protective role, as the mean weight of the women in the study was below that of their male counterparts (*P *< 0.0001) (Buckey et al., 2018). The head‐down tilt bed rest (HDTBR) analogue of microgravity does not appear to produce ocular changes consistent with SANS, suggesting the loss of tissue compressive forces in reduced gravity as part of the aetiology of SANS and limiting terrestrial investigations of the problem. Data from recent studies utilizing the delivery of partial artificial gravity during flight with equipment in the Japanese Kibo Laboratory provide evidence that microgravity induces retinal cellular changes, which may, in turn, affect the integrity of the blood–retinal barrier, visual acuity and the risk of developing late retinal degeneration (Mao et al., 2018). A 2018 mouse study performed by the Kibo facility using brief periods of 1 *g* artificial gravity generated by centrifugal force resulted in a reduction in apoptosis of retinal vascular endothelial cells, when compared to spaceflight controls (Mao et al., 2018). These findings suggest a possible oculo‐protective function of brief periods of induced gravity.

**TABLE 1:** Effects of spaceflight on organ systems critical to human performance.

Organ/system	Clinical effect observed	Explanation (recommendations where available)	Evidence source (terrestrial vs. space, animal vs. human, male ♂ vs. female ♀ vs. both ⚥ vs. not specified)
Eye	Hyperopic shift	Globe flattening (Buckey et al., 2018; Lee et al., 2018) o Female sex may be protective	Space, human, ⚥
	Optic nerve oedema	Cephalad fluid shifts and cerebrovascular congestion o LBNP Cerebral lymphatic congestion Elevated CSF pressure in the subarachnoid space (Lee et al., 2020)	Space, human, ⚥
	Nerve fiber layer infarcts (cotton wool spots)	Cephalad fluid shifts and cerebrovascular congestion o LBNP o Partial gravity with the Japanese Kibo apparatus may serve a protective role by preventing spaceflight‐induced retinal apoptosis of vascular endothelial cells and retinal protein expression (Mao et al., 2018)	Space, human/animal, ♀/♂/⚥
Neurovestibular	***Early motion sickness*** ***Space motion sickness***	Delayed vestibular adaptation to microgravity, possibly more pronounced in women (Reschke et al., 2014) due to the lower proportion of myelinated axons in the vestibular nerve (Moriyama et al., 2007) and relative size of inner ear organs (Sato et al., 1992) Vestibular disorders overall more common in women terrestrially (Neuhauser et al., 2005; von Brevern et al., 2007) o No clear link to sex in testing intended to simulate microgravity (Harm, 1990; Reschke, 1990)	Space/terrestrial, human, ⚥
	Speech‐frequency hearing impairment	History of high‐decibel noise exposure (Albera et al., 2010; Hoffman et al., 2016; Homans et al., 2016); no sex difference demonstrated in spaceflight environment (Buckey et al., 2001) o Enhanced hearing protection	Terrestrial, human, ♂/⚥
	Compromised balance postflight	Decreased demand on postural muscles (Holt et al., 2016; Lee et al., 2014) o Variable effects seen with treadmill, ARED, LBNP (Macaulay et al., 2016)	Space/terrestrial, human, ♀/⚥
Muscle	Loss of lower extremity lean muscle mass	Decreased demand on lower body muscles for mobility (Holt et al., 2016; Lee et al., 2014; Schneider et al., 2016) o Partial mitigation seen with treadmill, ARED, LBNP	Space/terrestrial, human, ♀/⚥
Bone	***Increased bone resorption markers inflight*** (Smith et al., 2003, 2014; Zwart et al., 2007)	Decreased loading force Short‐term reduced energy availability increases resorption markers in women but not men (Papageorgiou et al., 2017)	Space/terrestrial, human, ♂/♀/⚥
	Loss of areal BMD in femoral shaft and hip (Morgan et al., 2014; Smith et al., 2014; Zwart et al., 2007)	Decreased loading force o Treadmill, ARED, LBNP (Zwart et al., 2007)	Space/terrestrial, human, ♀/⚥

*Note*: In bold italics: effects more pronounced in women. Abbreviations: ARED, advanced resistive exercise device; BMD, bone mineral density; CSF, cerebral spinal fluid; LBNP, lower body negative pressure.

Terrestrial sex‐related differences in vision processing appear to be heterogeneous. There is a well‐established male preponderance of colourblindness (red–green subtype) (Abramov et al., 2012; Bimler et al., 2004). In a large‐scale terrestrial study, males tended to outperform females in simple visual reaction time, visual acuity, visual backward masking, motion direction detection, biological motion and the Ponzo illusion (Shaqiri et al., 2018), but these effects have not been examined in space. Unfortunately, the aforementioned studies did not control for the potential confounding factor of age. No significant sex differences have been noted for parameters such as contrast detection threshold, visual search, orientation or discrimination, among others (Schrauf et al., 1999). It is unclear if these visual processing elements are impacted by exposure to microgravity.

## 3 NEUROVESTIBULAR, AUDITORY AND SENSORIMOTOR

Functional deficits in spatial disorientation and proprioception on entry into weightlessness may impact mission completion by crewmembers of either sex if adaptive mechanisms are impaired. Anatomical factors, including fewer myelinated axons (Moriyama et al., 2007) and smaller inner ear structures (Sato et al., 1992), may explain the female predisposition to developing vertigo and other vestibular disorders on Earth (Neuhauser et al., 2005; von Brevern et al., 2007). Despite this terrestrial bias, no statistically significant sex‐based differences in motion sickness incidence were observed in testing intended to simulate the spaceflight environment; additionally, the phase of the menstrual cycle did not impact susceptibility among female subjects (space environmental impacts summarized in Table 1) (Harm, 1990; Reschke, 1990). Data mined from the NASA Lifetime Surveillance of Astronaut Health database indicate that female Shuttle and International Space Station (ISS) astronauts more frequently reported symptoms of space motion sickness and early motion sickness than their male crewmates (Reschke et al., 2014).

Hearing sensitivity declines with age across both sexes, even when controlling for a history of high‐decibel noise exposure (Albera et al., 2010; Homans et al., 2016), but appears to degrade more rapidly in men than in women (Hoffman et al., 2016). The prevalence of speech‐frequency hearing impairment among all adults in the USA has declined nearly 2% in the last two decades with enhanced public initiatives aimed at preserving auditory health. Sex differences persist, but there is no evidence to indicate an associated bias in the effects of occupational noise on astronaut crews (Buckey et al., 2001). Dedicated studies should be performed to closely examine sex‐specific effects in the spaceflight environment (Buckey et al., 2001).

Women tend to outperform men in tactile spatial acuity and global somatosensory perception (Kimchi et al., 2009), but the reverse is true for spatial orientation‐related tasks (Collins & Kimura, 1997; Moffat et al., 1998; Pena et al., 2008; Voyer & Hou, 2006). There appears to be an inverse relationship between tactile perception and finger size, such that smaller fingers tend to correlate with enhanced tactile acuity; this is thought to be a function of the higher density of sweat pores and Merkel receptors with decreasing fingertip surface area: one model demonstrated that ‘tactile thresholds increase at a rate of 0.25 mm/cm^2^ fingertip surface area (95% confidence interval: 0.11–0.40 mm/cm^2^)’ (Peters et al., 2009). These effects should be examined in the spaceflight environment to identify any potential sex‐dependent somatosensory adaptive mechanisms specific to weightlessness.

## 4 BEHAVIOURAL HEALTH, COGNITION AND RESILIENCE

Behavioral health‐related concerns are of critical importance in the aerospace environment, where smooth interpersonal relationships that permit inter‐reliance among team members are essential to ensure mission success. Extensive research over the last several decades has illuminated many ways in which psychological stress – from physiological changes, isolation from loved ones, and an awareness of the austere and dangerous nature of the spaceflight environment – poses challenges for psychological wellbeing.

In terms of cognition, terrestrial studies reveal several sex‐based differences, but the relevance of these in the spaceflight environment remains unknown. Memory processing in the amygdala is strongly linked to emotionally charged stimuli, especially visual images. Processing of identical emotional stimuli appears to occur preferentially in the left amygdala in females, but on the right in males (Canli et al., 2002), though the clinical relevance of this distinction is unclear (Cahill et al., 2001, 2004; Canli et al., 2002). No sex‐based predisposition to radiation‐related neurocognitive deficits has been identified (Asai & Kawamoto, 2008; Greene‐Schloesser et al., 2013; Hladik & Tapio, 2016).

Anxiety and depression, two widespread mental health conditions, affect both men and women in the terrestrial population. Anxiety disorders are reportedly more prevalent in women (Hantsoo & Epperson, 2017). Age, education level and comorbidities strongly influence the incidence of these conditions, rendering the findings of most terrestrial studies only marginally applicable to the astronaut population. Depression poses a particularly serious threat to crew cohesion, should it manifest in‐mission, particularly given the interpersonal and social ramifications of mood disorders, which may become magnified in a remote and confined setting (Yin et al., 2023). Among women, depressive episodes often follow familial or other interpersonal relationship stressors, whereas in men, episodes are more often triggered by financial or work‐related concerns (Altemus et al., 2014). No direct correlation has been found between the effects of radiation‐related changes in oestrogen and testosterone secretion and the risk of mood disorders. Gonadal tissue in both men and women exhibits similar susceptibility to radiation toxicity (Narendran et al., 2019). As in several other subject areas, sex‐based differences in cognitive and behavioural adaptation of astronauts to the spaceflight environment have yet to be well characterized, though the ‘select‐in’ nature of the astronaut candidate application process certainly renders these factors important.

Teamwork and communication, among the most critical elements as mission duration and distance from Earth increase, are two areas in which women categorically shine. In the medical setting, recent studies reveal large‐scale reductions in patient mortality and hospital readmission rates, along with improved surgical outcomes, for female physicians and surgeons compared to their male counterparts (Tsugawa et al., 2017; Wallis et al., 2017); the effectiveness of lifestyle changes, behavioural modifications and overall patient engagement also seems to benefit from a female physician's leadership and guidance (Baumhäkel et al., 2009; Roter et al., 2002). These conclusions are not intended to have a divisive effect in the professional setting, as both men and women certainly have served as powerful and effective teammates in the spaceflight setting, but rather, to emphasize the complementary nature of balanced and diverse crew composition – a proven key to a team's long‐term agility and success in many sectors (Rock and Grant, 2016).

Psychological resilience, a close cousin of grit (the passion and perseverance for long‐term goals; Schimschal et al., 2021), is the subject of extensive ongoing research. The definition of resilience is varied depending on the research area. Perhaps the most applicable definition for astronauts comes from military research publications where resilience is defined as the personal qualities that enable one to thrive in the face of adversity (Connor & Davidson, 2003). Resilience may be the single most important factor in ensuring the success of a lengthy interplanetary mission. Communication style, often linked to gender, is a primary driver of one's subconscious method for managing chronic stress. A number of large‐scale studies have uncovered the role that a strong verbal coping mechanism plays in augmenting resilience (Tamres et al., 2002). In the spaceflight setting, these effects may be further enhanced by the reduced susceptibility of female brain tissue to GCR‐related damage that has been demonstrated in the murine model (Krukowski *et al.*, 2018). While additional investigation is warranted, the results of these and other studies suggest an overwhelmingly beneficial effect of the presence of female crewmembers in high‐pressure team environments such as deep space.

## 5 SLEEP

Sleep plays a vital role in optimizing human health and warding off physiological and behavioural disorders. Circadian rhythm (CR), the body's ‘24‐hour internal clock’, correlates periods of alertness and sleepiness with patterns of light and darkness perceived by the retina over the course of a day (Reddy et al., 2020). Circadian misalignment compromises sleep quality and duration, which can predispose individuals to a wide spectrum of diseases, including obesity, heart disease and cancer (Noguti et al., 2013). Sleep is also instrumental in maximizing performance on cognitive tasks, ranging from executive and psychomotor functions to emotional regulation and maintenance of interpersonal relationships (Albornoz‐Miranda et al., 2023; Jones et al., 2022; Yamazaki & Goel, 2020). During spaceflight operations, the impact of sleep on alertness, reaction time and cognitive performance may mean the difference between life and death (Ellis, 2000).

Physiological effects of altered sleep patterns differ between sexes, particularly in the setting of chronic sleep restriction, defined as ‘habitual sleep durations that are less than 7 hours, but more than 4 hours, a night’ (Magee et al., 2010). Over time, sleep loss induces greater increases in leptin, a satiety hormone, and enhanced activation of pro‐inflammatory cytokines (i.e., interleukin (IL)‐6 and tumour necrosis factor α) in women, relative to men. On Earth, sleep disruption in women increases levels of inflammatory markers (i.e., hs‐CRP) and pro‐coagulants (i.e., von Willebrand factor), which may increase the risk of cardiometabolic disease and the overall rate of CV events including strokes (Previtali et al., 2011). Long‐term sleep deprivation has been tied to depression and weight gain among both sexes, with no clear sex‐linked obesity trend (Koren et al., 2016). Women tend to exhibit shorter sleep latency and overall greater sleep efficiency compared to men, possibly protecting women from fatigue‐associated mental health and body composition changes. Hypoxia associated with the spaceflight environment may exacerbate sleep issues. In a short, 10‐day hypoxia with confined bed rest study in women, a negative impact on sleep macrostructure, microstructure and respiratory functioning was observed (Van Cutsem et al., 2022). African‐American females may be more resistant to weight gain with long‐term sleep deprivation due to increasing circulating adiponectin levels with chronic sleep deprivation, while circulating concentrations decrease in Caucasian females. Chronic sleep deprivation does not appear to significantly alter circulating adiponectin levels in men. The differential effects of sleep deprivation on male and female mental health in microgravity are understudied and should be emphasized in future research.

Flight physicians offer medications (i.e., zolpidem, modafinil, etc.) to crewmembers to promote either sleep or wakefulness as dictated by mission requirements (Barger et al., 2014; Crucian et al., 2016). Between 71% and 78% of crew members use such medication to initiate sleep at some point in‐mission (Albornoz‐Miranda et al., 2023). The effects of long‐term use of these medications on Earth can include mood dysregulation and decreased medication effectiveness over time (Hart et al., 2003; Van Camp, 2009), while research is ongoing to understand these effects in the spaceflight environment (Boschert et al., 2019).

## 6 CARDIOVASCULAR

CV pathology is the leading cause of death worldwide. Women develop CV disease approximately 10 years later and have greater CV morbidity and mortality relative to men, perhaps owing to the lack of optimal preventative strategies, diagnostics and therapies tailored to female physiology (Platts et al., 2014). This has implications for women terrestrially, as well as in space. However, it should be noted that the CV conditioning required of both male and female astronauts premission as well as in‐mission likely provides a protective effect. This element is more representative of athlete physiology than that of the typical Earth‐based population.

There are well‐documented cephalad fluid shifts that occur in the first 48 h after an astronaut has entered microgravity (space environmental impacts summarized in Table 2); the haemodynamic effects of this shift include changes in cardiac stress as well as transient hypovolaemia‐related complications following return to Earth (Hargens & Richardson, 2009). The prevalence of orthostatic intolerance (OI) upon return to the 1 *g* environment may be higher among female astronauts compared to males, but is confounded by several factors, including a greater propensity for females to report symptoms than their male counterparts; few studies have targeted women and minority ethnic groups to better understand any pertinent haemodynamic differences. For reasons unknown, female astronauts tend to have a greater relative plasma volume loss than men while adapting to weightlessness. Terrestrially, women appear to have a more active parasympathetic system, higher oestrogen levels, and a lower centre of gravity, which may predispose them to OI (Cheng et al., 2011). OI appears to be refractory to midodrine among female astronauts, unlike their male crewmates (Platts et al., 2014), but the protective role of exercise in improving OI, preserving cardiac volume and increasing cardiac mass during spaceflight is more pronounced in women (Pavy‐Le Traon et al., 2007). While these effects are typically short‐lived, resolving after several days at 1 *g*, the performance‐related implications for crews landing on Mars after many months in transit justify further investigation. The compounding effects of radiation‐associated cardiotoxicity on OI, exertional capacity, and overall ability to perform mission tasks after several months transiting deep space are currently difficult to predict (Boerma et al., 2016).

**TABLE 2:** Cardiovascular and pulmonary effects of exposure to the spaceflight environment.

Organ/System	Clinical effect observed	Explanation (recommendations where available)	Evidence source (terrestrial vs. space, animal vs. human, male ♂ vs. female ♀ vs. both ⚥ vs. not specified (NS))
Heart	***Generalized hypovolemia (loss of 1–2 litres of intravascular volume) inflight***	Space adaptation o Greater relative plasma volume loss in women (Hargens & Richardson, 2009; Hargens & Vico, 2016)	Space, human, ⚥
	***Transient OI*** ^a^ ***on return to Earth***	Re‐entry effects o Midodrine (refractory in women) (Cheng et al., 2011; Platts et al., 2014) o Exercise (Pavy‐Le Traon et al., 2007) Radiation‐associated cardiotoxicity and concomitant reduction in exertional capacity	Terrestrial, human, ♀/♂/NS
Blood vessels	***Internal jugular vein clot***	Venous stasis and retrograde flow in cervical plexus Possible role of COC with theoretical resultant hypercoagulability (Plu‐Bureau, Maitrot‐Mantelet, et al., 2013; Previtali et al., 2011; Ronca et al., 2014); no change in risk of MI	Space, human, ⚥
	Decreased vascular compliance	Radiation toxicity Endothelial microtears due to aberrations in flow (Xu et al., 2018)	Space, human, ⚥
Lungs	Pulmonary fibrosis	Radiation (Townsend et al., 2012) Chronic exposure to irritants such as lunar regolith (Johansen et al., 2018; Prisk, 2011, 2019) o Men may be at higher risk of regolith‐linked pulmonary fibrosis than women	Terrestrial, human, ♀/♂/⚥
	***Pulmonary hypertension***	Proposed hormonal role to explain elevated risk in women (Sathish et al., 2015)	Terrestrial, human, ♀/♂/⚥
	Lung cancer	Various terrestrial oncogenic factors; role of space radiation proposed Higher incidence of IR‐related cancers in women than in men (Narendran et al., 2019) Improved 5‐year survival rates among female lung cancer patients who undergo chemotherapy, versus men (Carey et al., 2007)	Terrestrial, human, ⚥

*Note*: In bold italics: effects more pronounced in women.

^a^ Orthostatic intolerance: OI is defined as a decrease in systolic blood pressure (BP) >20 mmHg, or a decrease in diastolic BP of >10 mmHg within 3 min of transitioning from supine to an upright position. Abbreviations: COC, combined oral contraceptive; IR, ionizing radiation; MI, myocardial infarction.

Terrestrially, while there is no overt evidence of increased risk of myocardial infarction in women using combined oral contraceptives (COC), the increase in venous thromboembolism risk in this patient group is well‐documented (Jain et al., 2020; Plu‐Bureau, Hugon‐Rodin, et al., 2013; Previtali et al., 2011). Newer COC options with decreased levels of exogenous oestrogen may reduce risk but have not been studied as thoroughly as the older COC preparations. In female astronauts, the use of COCs was related to lower circulating albumin, and higher transferrin and elevated markers of inflammation – in association with higher blood viscosity could contribute to an increased risk of venous thromboembolism risk (Zwart et al., 2022). In the spaceflight environment, many female astronauts eliminate the logistical and resource‐related challenges of menstruation inflight by opting for a COC regimen that suppresses menstruation altogether.

## 7 PULMONARY

Several sex‐based anatomical differences characterize the architecture of the respiratory tract. In the upper airway, increased retropalatal collapsibility is thought responsible for the higher prevalence of sleep‐disordered breathing conditions such as obstructive sleep apnoea (OSA) seen in men. While sleep‐disordered breathing tends to improve in short‐duration exposure to the spaceflight environment (Dinges, 2001), the known hypoxia and cardiac strain posed by OSA, along with its many detrimental effects on attention and performance, render this condition a significant enough risk in the spaceflight setting to warrant discussion. Testosterone levels appear to correlate directly with neck circumference as well as the incidence of OSA (Rowley et al., 2002). Higher fat deposition in the neck and upper torso is also seen among postmenopausal women, but rates of OSA do not parallel this trend (Wimms et al., 2016).

Anatomically, men have significantly larger ribcage cross‐sectional diameters and up to 9% longer diaphragms relative to women, accommodating higher lung volumes at various phases of the respiratory cycle (LoMauro & Aliverti, 2018). Women are known to have lower total alveolar number and surface area, presumably due to overall smaller body size, as alveolar number per unit area and volume typically remain constant regardless of sex. Men's ribs are oriented more horizontally than women's, and the slight, consequent angular difference in outward rib movement with chest expansion as seen in women appears to better accommodate a gravid uterus (Bellemare et al., 2003). These angular differences may lend a mechanical advantage to the intercostal muscles of women and explain the disproportionate increase in diaphragmatic breathing seen in men relative to women (Torres‐Tamayo et al., 2018). Changes in these morphological parameters in microgravity may have sex‐specific implications.

Conditions such as lung cancer and pulmonary fibrosis vary epidemiologically between sexes. The prevalence of pulmonary fibrosis is higher among men (Sathish et al., 2015; Townsend et al., 2012), with male:female incidence ranging from 1.4:1 to 2.1:1. Conversely, pulmonary hypertension affects a disproportionately large number of women, as the incidence of all subtypes of pulmonary hypertension ranges from 1:2 to 1:4 (male:female). Ground‐based studies reveal rising lung cancer mortality rates among women in recent years, while this parameter has remained stable among men. Non‐smoker females are three times as likely as their male counterparts to be diagnosed with lung cancer, suggesting a possible hormonal element to pulmonary oncogenesis – although this has yet to be investigated in microgravity (Narendran et al., 2019). IR‐related lung cancer and treatment responses appear to be tightly linked to sex (Carey et al., 2007).

Pulmonary pathology is of special concern on upcoming missions to the Moon, for which repeated exposure to lunar regolith is anticipated (Johansen et al., 2018; Prisk, 2019), and terrestrial studies pose the question of whether male astronauts may be at higher risk of developing this condition than their female crewmates. A summary of known space environmental impacts is found in Table 2. Further work in this area is needed in order to elucidate these risks (Prisk, 2011).

## 8 IMMUNOLOGY AND HAEMATOLOGY

During the Apollo program, 15 of 29 male astronauts reported bacterial or viral infections during or immediately after return. The most famous incident was on Apollo 13, when one astronaut developed a severe opportunistic *Pseudomonas aeruginosa* infection, a condition usually seen only in immunosuppressed patients (Hawkins, 1975). It should be noted that the emergent return undertaken as a part of the Apollo 13 mission, during which the crew was isolated in cold temperatures in one capsule, carried with it a requirement for prolonged use of urine collection devices; under these conditions, a woman might have had the same susceptibility to developing a urinary infection. Understanding and anticipating adaptations involving the immune system pose unique challenges to the field of Space Health (Kennedy et al., 2014). Women have stronger immune responses to both infections and vaccination than men. Paradoxically, the stronger immune response comes at a steep price: the high incidence of autoimmune disease among Earth‐bound women (Moulton, 2018).

In the USA, approximately 5% of the population is afflicted with an autoimmune disease; of those individuals, approximately 75% are women (Whitacre, 2001). All astronauts are screened for known autoimmune disorders for which space travel may pose an increased risk to the individual. While only approximately 11% of current astronauts are women, all of them are healthier and more physically fit than the general population. As the number of women with flight time increases, we will begin to understand the sex‐specific differences in autoimmune conditions associated with spaceflight. Aggregated information regarding the space environment's impact on the immune system can be found in Table 3.

**TABLE 3:** Immunological effects of the spaceflight environment.

Cellular/tissue‐based immune response observed in spaceflight	Effects	Explanation	Evidence source (terrestrial vs. space, animal vs. human, male ♂ vs. female ♀ vs. both ⚥ vs. not specified)
Thymus (source of T‐cells) atrophies	Significant atrophy of thymus in spaceflight (Horie et al., 2019)	Artificial gravity significantly mitigates the reduction of thymus size	Animal, ♀ (Horie et al., 2019)
Adaptive immune response in human T‐cells	Early T‐cell expression is regulated by miRNA; no difference is seen in men versus women (Hughes‐Fulford et al., 2015)	Human cells were flown and half were in the 1 *g* onboard section of cubic incubator regulation of 1 *g* samples showed miRNA differences	Human immune cells, ⚥ *n* = 8 spaceflight, versus *n* = 8 onboard 1 *g* inflight *n* = 26 simulated microgravity versus 1 *g* controls on Earth
Adaptive immune response in humans	Reduced T‐cell response persisted in long‐term flight in humans; no significant immunological difference between men and women (Crucian et al., 2015)	WBC and granulocyte numbers increased inflight while lymphocyte and monocytes were unaltered. NK cells elevated late inflight	Human, ⚥ 18 ♂ and 5 ♀ crew
Countermeasure‐based improvements in stress and immune system dysregulation on ISS	Major physiological improvements seem to have been initiated approximately 2012, a period coinciding with improvements onboard ISS; no significant immunological difference between men and women (Crucian et al., 2020)	Other factors correlated with improvements: personal communication, exercise equipment and protocols, food quality and variety, nutritional supplementation, and schedule management	Human, ⚥ 31 ♂ and 8 ♀ crew

Abbreviations: ISS, international space station; miRNA, micro ribonucleic acid; NK, natural killer; WBC, white blood cells.

Chang et al. demonstrated that spaceflight impairs antigen‐specific tolerance induction in female mice, resulting in increased inflammatory cytokines when compared to ground controls (Chang et al., 2015). This study was the first to show that immune tolerance can be impaired in spaceflight, leading to inflammatory responses and possible autoimmune disorders inflight. Many mice studies include only females due to increased aggressive behaviour in male rodents. In the Bion M‐1 spaceflight, only 36% (16 out of 45) of male mice launched survived (Andreev‐Andrievskiy et al., 2014). A recent ground‐based simulated GCR exposure (GCRsim) (50cGy) with housing differences in 6‐month‐old male and female mice demonstrated that the GCRsim drove early observed changes in immune cell populations, while social isolation housing and hindlimb unloading drove later changes in immune cell populations (Rienecker et al., 2023). The authors additionally reported that female mice were largely resistant to the deficits observed in the male mice.

In the spaceflight environment, early T‐cell activation is suppressed via the Rel/nuclear factor κB signalling through changes in mRNA expression. These effects are mediated by dysfunctional early immune activation, wherein IL‐2 expression is decreased; full, robust immune activation is not achieved in this setting due to a lack of interleukin receptor subunit IL‐2Ra mRNA and protein transcription (Chang et al., 2012). To date, this scientific finding has been observed only in female mice. Immune dysfunction among humans in spaceflight is seen in the form of rashes and other hypersensitivity reactions that tend to persist for longer periods than they normally would on Earth. Latent viral reactivation has been observed in astronauts on both the Shuttle and ISS, but to date, there have been no sex‐based differences reported preflight, inflight or post‐flight (Crucian et al., 2018; Mehta et al., 2017; Rooney et al., 2019). In a recent ISS study, crewmembers with the highest cardiorespiratory fitness experienced a 29% reduced risk of latent viral reactivation. Viral reactivation rates were highest in crew with low cardiorespiratory fitness both before and after flight (Agha et al., 2020). Additionally, higher preflight upper body muscular endurance was associated with a 39% reduction in reactivation risk, a longer time to viral reactivation when it did occur, and lower peak viral DNA concentrations for Epstein–Barr virus (EBV) and varicella zoster virus (VZV). No sex‐linked difference in protection from reactivation between fit men and fit women for EBV and VZV has been observed. There was no fitness‐related protection from cytomegalovirus (CMV) reactivation; however, only 59% of the crew tested seropositive for CMV. In the past 8 years, lower incidence of viral reactivation has been noted, corresponding with an increase in resupply frequency, enhancement of personal communication, improvement of exercise equipment and food quality, and betterment of schedule management (Crucian et al., 2020).

Sex differences are seen in the novel coronavirus disease 2019 (COVID‐19) pandemic. Many had predicted that the next ‘100‐year pandemic’ would be caused by an influenza A virus, as in 1918; instead, the current pandemic is caused by a novel β‐coronavirus, the severe acute respiratory syndrome coronavirus 2 (SARS‐CoV‐2). According to the World Health Organization, as of 19 July 2023, there have been in excess of 768 million confirmed cases and over 6.9 million deaths worldwide from COVID‐19 (2023). As was seen in the 1918 influenza pandemic, men are at greater risk of more severe COVID‐19 outcomes than women, with both sex (i.e., biological differences) and gender (i.e., sociocultural and behavioural differences) playing fundamental roles (Morgan & Klein, 2019). Similar numbers of confirmed SARS‐CoV‐2 cases have been reported in China and Europe between men and women. However, the severity of COVID‐19, as measured by hospitalizations, admissions to intensive care units, and rates of fatality, has consistently been two‐fold greater for men than women (Gebhard et al., 2020).

Principal spaceflight‐associated haematological concerns centre around anaemia and bone marrow suppression. As a function of acute dehydration and plasma volume changes, there is a transient elevation in haematocrit, which reduces erythropoietin secretion by the kidney (Alfrey et al., 1996; Rice & Alfrey, 2000). The anaemia developed by astronauts appears to resolve after initial adaptation to microgravity (Kunz et al., 2017). Homeostatic mechanisms appear to trigger rebound erythropoietin secretion on return to Earth in response to the overall reduced haematocrit observed in the postflight phase (Buckey, 2006). This may contribute to the observed postflight orthostatic hypotension. More focused study is necessary to identify if any sex‐linked predisposition to haematological derangements exists with exposure to microgravity.

IR is known to affect the immune and haematological systems. With total acute doses approaching 1–2 sieverts (Sv), a 50% decrease in circulating lymphocytes and neutrophils is seen, while global bone marrow suppression is observed following doses over 2 Sv (Robbins, 1994). Exposure to GCRsim (50 cGy) caused changes in circulating immune cell populations, and continued immune cell changes were observed to be caused by isolation due to housing conditions in male mice, while female mice were somewhat resilient to deficit developments under the same conditions (Rienecker et al., 2023). Potential sex differences in this area would benefit from further investigation.

## 9 MUSCULOSKELETAL

The musculoskeletal systems of men and women differ, with men generally having greater muscle and bone mass due to extended periods of growth during puberty (Almeida et al., 2017). It is well established that the human musculoskeletal response to unloading (summarized in Table 2) is highly variable among individuals, no matter the sex (Clark et al., 2006; Vico et al., 2000), but age may play a role in the observed compartment‐specific bone loss (Coulombe et al., 2021). Since the last review on the effects of sex and gender adaptations to spaceflight (Ploutz‐Snyder et al., 2014), few human or animal studies have been powered to assess potential sex differences in musculoskeletal adaptation to the spaceflight environment (Cavanagh et al., 2016).

In a direct assessment of sex differences during 30‐day HDTBR with identical twins, there were no significant pre‐ to post‐muscular changes in control or exercise + lower body negative pressure (LBNP) randomized female twins. The exercise + LBNP countermeasure partially attenuated losses of leg lean mass (LLM), strength and endurance in the male twins (Schneider et al., 2016). The exercise + LBNP countermeasure did not protect male or female subjects’ rail walk abilities, but attenuated loss of balance in male subjects. In the all‐female HDTBR study (Women International Space Simulation for Exploration; WISE), a combined resistive and aerobic exercise protocol protected participants against the loss of lower limb muscle strength and endurance, LLM and paraspinal muscle structure and function, while a nutritional countermeasure alone was not effective (Holt et al., 2016; Lee et al., 2014). In Wistar rats, 14 days of hindlimb suspension resulted in the maintenance of muscle function and strength in females compared to males, which was associated with a lesser deconditioning during disuse in females (Mortreux et al., 2021), while women showed more pronounced losses in jumping power and knee extension strength in the AGBRESA study compared to men (Kramer et al., 2021).

In an analysis of 42 ISS astronauts (33 men and nine women) on long‐duration missions (49–215 days), areal bone mineral density (BMD) and bone biochemical marker changes in response to spaceflight were the same for men and women using either of the available resistance exercise devices (Smith et al., 2014). Men and women using the advanced resistive exercise device (ARED) did not have the typical decrease in areal BMD noted post‐spaceflight. Similarly, a *post hoc* analysis of control subjects (50 men and 24 women) from five studies indicated that sex did not substantially impact the bone response to HDTBR (Morgan et al., 2014). These results suggest that there is no sex difference in the rate of bone loss with unloading. Recent data suggest that the elevated CO_2_ exposure experienced on the ISS is not exacerbating the bone resorption effect observed inight and with HDTBR studies (McGrath et al., 2022). The identical twins HDTBR study reported similar trends in bone marker changes in all control subjects (Smith et al., 2003; Zwart et al., 2007); however, exercise/LBNP countermeasures attenuated the increase in bone resorption markers in male subjects (Smith et al., 2003). The potential explanations for the sex differences include not controlling for the cyclic changes in reproductive hormones in the female subjects (Zwart et al., 2007) and the higher baseline fitness and potential ability to generate greater peak ground reaction forces in the male subjects (Smith et al., 2003). The difference in intrinsic bone metabolism between age groups examined in the identical twins HDTBR study (women 24 ± 3 years and men 27 ± 5 years) versus the ISS and *post hoc* HDTBR controls (women 35 ± 9 years and men 33 ± 8 years) potentially explains some of the differences in these reports.

Unique to female astronauts are decisions surrounding the control of reproductive function. NASA's astronaut population has exercised autonomy in selecting an individualized strategy for menstrual suppression, hormonal contraception (HC), or other hormonal therapy indications. Terrestrially, post‐menopausal hormone therapy is beneficial to the musculoskeletal system (Eastell et al., 2019; Rozenberg et al., 2020), but there is conflicting evidence regarding the impact of HC use during the pre‐ and peri‐menopausal periods (Gambacciani et al., 2006; Hartard et al., 2006; Scholes et al., 2011; Weaver et al., 2001). Decreased BMD has not been associated with COC containing 0.030–0.035 mg of ethinyl oestradiol (Berenson et al., 2004) and has traditionally been the choice for astronauts inflight (Jain et al., 2020; Jennings & Baker, 2000). The combination of exercise and use of COC may result in reduced areal BMD (Hartard et al., 2006; Weaver et al., 2001), but this impact may depend on age, progestin and dose of ethinyl oestradiol. Bone site, duration of use and route of administration alter the impact of long‐acting, reversible contraception use on BMD (Beerthuizen et al., 2000; Modesto et al., 2015; Monteiro‐Dantas et al., 2007; Nappi et al., 2012). Limited data on HC use and skeletal muscle health exist (Hansen et al., 2009, 2011).

Important sources of musculoskeletal injury include fracture, ligament tears, muscle tears and back pain due to the expansion and/or degeneration of the vertebral disk (Belavy et al., 2016). Rotator cuff injuries and other joint injuries are common during training and flight (Fincke et al., 2005; Scheuring et al., 2009; Stirling et al., 2019). The healing of both hard and soft tissues is delayed in microgravity, with fibroblasts, muscle cells and the extracellular matrix sensitive to altered gravitational forces (Blaber et al., 2014; Liu & Wang, 2008; Saito et al., 2003). There is currently no indication of any sex difference in the number of injuries or propensity to recover from injury associated with spaceflight.

Beyond the risk of disuse, IR exposure will influence musculoskeletal adaptation to unloading. Currently, little is known about the combined effects of weightlessness and radiation exposure at space‐relevant doses, as it is difficult to simulate both conditions simultaneously for in vivo studies. In male and female mice, acute, high doses (2+ Gy) of IR cause trabecular bone loss, increased number of osteoclasts (Alwood et al., 2017; Hamilton et al., 2006; Kondo et al., 2010; Kumar et al., 2022; Willey et al., 2008; Wright et al., 2015) and apoptosis of bone marrow stem cells (Kondo et al., 2010). Lower acute and continuous IR doses (<0.5 Gy) result in increases or no changes in trabecular bone volume (Alwood et al., 2017; Bandstra et al., 2009; Bokhari et al., 2019; Karim & Judex, 2014; Yu et al., 2017). However, single IR type and energy exposure are not the same as GCR exposure.

Some of the interindividual variability in response to spaceflight and analogues may be related to age‐ and sex‐specific hormonal factors. Whether the statistical differences in the musculoskeletal system translate into clinical or performance decrements is unclear. The extent to which biological sex contributes to interindividual variability in altered gravity and IR requires further investigation in order to develop effective musculoskeletal countermeasures for use during exploration‐class missions. Intrasex variability in response to unloading and exposure to IR is substantial and should be further explored.

## 10 NUTRITION AND ENERGY BALANCE

While no data are yet available regarding differences between what male and female astronauts eat in space, the data are being collected more systematically with the change from a food frequency questionnaire to an intake tracking app for ISS astronauts. Historically, dietary intakes during spaceflight since the Apollo era (except for Skylab and specific flights to Mir) had been in the range of 70–80% of predicted requirements (Smith et al., 2005). During Skylab and on some European flights to Mir, astronauts participated in metabolic experiments utilizing the consumption of eucaloric diets that were balanced and controlled – thus these crew members met their recommended daily energy intake (Drummer et al., 2000; Leach & Rambaut, 1977; Rambaut et al., 1977). Many aspects of longer‐duration missions have evolved on the ISS, including the reformulation of many food items, the inclusion of international partner agency foods, and the passage of lessons learned between crews leading to recent observations of astronauts meeting recommended dietary intakes and maintaining body mass (Smith et al., 2005, 2012).

Many astronauts still lose 2–5% of their initial body mass while inflight, a change which may occur primarily during the first month and remain stable over the remainder of the mission (Zwart et al., 2014). This continued initial loss is likely due to a reduced intake in the first days of a mission related to space motion sickness (Heer & Paloski, 2006; Lackner & Dizio, 2006; Reschke et al., 1994; Seddon et al., 1994). Space motion sickness effects typically pass after a few days but the reduced dietary intake has been observed well beyond the first week (Lane & Smith, 1999). Anecdotal reports have been published indicating changes in taste during flight (Baranski et al., 1983; Olabi et al., 2002; Seddon et al., 1994). This anecdotal evidence has not been systematically followed up but could be related to impacts on olfaction related to the lack of convection in microgravity, competing odors in the small, confined, and closed volume of the ISS, higher CO_2_ concentrations, and use of recycled water on board the ISS (Baranski et al., 1983; Olabi et al., 2002; Seddon et al., 1994; Taylor et al., 2020). Another factor may be related to changes in GI function, whether in motility or absorption. Changes in nutrient absorption have not been reported beyond altered fuel homeostasis (Bergouignan et al., 2010, 2011; Blanc, Normand, et al., 2000; Stein & Wade, 2005; Stein et al., 2005; Zahariev et al., 2005), but in analogue missions (HDTBR and MARS‐500), GI motility has been explored. While confinement alone had no observable effect on GI motility (Roda et al., 2013), HDTBR increased mouth‐to‐caecum transit time (Lane et al., 1993).

Though it has been evaluated in HDTBR (Blanc et al., 1998) and short‐duration flight (Stein, Leskiw, et al., 1999) experiments, the basic energy needs of men and women during long‐duration flight, including expenditure during physical activity, require further study. Recent modelling assessments of total daily energy expenditure, oxygen consumption, carbon dioxide production and heat production during short‐ and long‐duration missions with and without ISS‐like exercise countermeasures included in the models demonstrated a relative difference of −5% to −29% for all female parameter estimates compared to males indicating a potential advantage for missions with more female crew members (Scott et al., 2023), and the need for more dedicated research. In the recent AGBRESA study, there were indications that simulated microgravity increased parameters related to iron metabolism in males but not females despite their loss of muscle mass, but were cautious in the interpretations due to the imbalance and low number of females recruited for the study (Horeau et al., 2022).

Existing exercise countermeasure programmes, along with physically demanding extravehicular activities, result in high‐volume energy expenditure. If food intake is not adjusted to match the total energy expenditure, a negative energy balance will ensue. Neuroendocrine and physiological adaptations occur with a prolonged negative energy balance to enable the body to conserve energy. In athletes, the adaptations to a negative energy balance are known to impact bone, muscle, reproductive function, CV function and performance, to name a few, with more extensive literature on females (Joy et al., 2014; Mountjoy et al., 2018; O'Leary et al., 2020). Recent reviews on nutrition, metabolism (Bergouignan et al., 2016) and the role of exercise in energy balance in space highlighted key knowledge gaps in multiple areas, irrespective of potential sex differences (Laurens et al., 2019). Importantly, microgravity, altered CR, and IR have the ability to affect food intake by altering hypothalamic activity, cytokine and orexigenic/anorexigenic hormone profiles (Ivanisevic‐Milovanovic et al., 1995; Mahapatra et al., 1988) and taste preferences (Bourland et al., 2000; Hunt et al., 1989), in addition to perturbing gut microflora.

The paucity of literature evaluating sex‐related differences in the impact of a negative energy balance on organ systems involved with performance has not changed appreciably since 2014. In athletic young adults, short‐term (5 days) reduced energy availability resulted in increased bone resorption and decreased bone formation markers in women but had no effect on bone turnover markers in men (Papageorgiou et al., 2017). Higher interindividual variability was seen among male participants due to the short‐duration perturbation, rendering the findings challenging to interpret. A longer duration or more pronounced negative energy balance is likely required to elicit a change in bone metabolism in men. Terrestrially, a long‐term energy deficiency is reported to impact bone health similarly in exercising women and men (Barrack et al., 2017; De Souza et al., 2014; Dolan et al., 2012; Leydon & Wall, 2002), with women potentially at higher risk for stress fracture (Wentz et al., 2011). A recent longitudinal metabolomic assessment in 4–6‐month duration ISS stays indicates that female astronauts potentially require longer readjustment phases post‐flight than male astronauts (Stroud et al., 2022). The potential impairment of crew performance due to negative effects on lean mass, bone mass, CV function and energy metabolism (Baek et al., 2008; Biolo et al., 2007; Blanc, Somody, et al., 2000b; Carpentier et al., 2018; Florian et al., 2015, 2016; Ihle & Loucks, 2004; Murphy et al., 2018; Phillips, 1994) highlights the need to increase our understanding of what amount of negative energy balance can be tolerated before mission success is compromised due to loss of physical performance and the need to coordinate energy intake with exercise regimens and extra‐vehicular activity energy expenditures.

## 11 ENDOCRINE

While there are many known terrestrial differences in endocrine function between sexes, this topic has been the focus of few spaceflight‐associated investigations (Stein, Leskiw, et al., 1999). Several endocrine processes involve profound enough sex differences terrestrially that they merit dedicated future study in space. (Note: Bone and muscle changes, though influenced by endocrine functions, have been addressed as a separate topic above.)

### 11.1 Thyroid and parathyroid functions

Thyroid and parathyroid functions are known to differ greatly between sexes, with a preponderance of related pathology arising in women. In addition to the heavy female burden of inflammatory and autoimmune thyroid conditions, thyroid cancer is nearly three times more common in women than in men (Rahbari, 2010). Exposure to IR increases thyroid cancer risk, and to a greater extent than most other solid organ cancers, though it requires further investigation in humans in the spaceflight environment. Ongoing, dedicated study in this area is advised, as it may reveal the need for targeted countermeasures such as collar shields or spacesuit modifications that could serve to mitigate the risk of disease.

### 11.2 Reproductive hormones

Major contributors to biological sex differences are reproductive organs as the balance of and cyclic changes in reproductive hormones impact the health of multiple systems throughout the body. Systematic evaluations of the physiological impacts of the spaceflight environment have not been conducted with these differences in mind (Ronca et al., 2014). Little regarding the impact of the spaceflight environment on reproductive tissues has been published since the last review on reproductive health adaptations to space (Ronca et al., 2014), with the exception of two recent reviews examining the reproductive hazards of space travel and gynaecological risk mitigation considerations for long‐duration spaceflight (Mishra & Luderer, 2019; Steller et al., 2020).

Albeit sparse, animal data from hindlimb unloading and hypergravity studies looking at the impact of altered gravity and radiation on female reproductive tissues indicate a lengthened oestrous cycle (Tou, 2004). Isolated cell cultures and histological examination of ovaries following flight indicated compromised follicle survival, decreased ovulation frequency, and reduced expression of oestrogen and progesterone receptors (Stoltz, 2019). Animal and cell data suggest numerous spaceflight‐associated effects on ovarian function (Sandler, 1978). The menstrual cycle has not adequately been accounted for and/or reported on with HDTBR studies, limiting insights into alterations in human ovarian function (Fortney et al., 1988; Wade & Baer, 2007; Zwart et al., 2007). In the WISE study, eight of 24 participants demonstrated shifts from eumenorrhoeic to oligomenorrhoeic cycle lengths with a lengthening of the follicular phase, delay of ovulation, and an overall reduction in circulating oestradiol and progesterone, which were independent of both the diet and exercise countermeasures (Wade & Baer, 2007). Menstrual cycle phase at the start of HDTBR was not controlled for, limiting interpretability. There are no formal inflight data regarding menstrual cycles available for premenopausal female astronauts, as most opt to use HC (Jain et al., 2020; Jennings & Baker, 2000). The pharmacology of HC in the spaceflight environment has not been investigated.

As with ovarian changes, serum testosterone begins to decrease with short‐term exposure to spaceflight and hindlimb unloading, with a period of adaptation observed in long‐duration exposure. With long‐term hindlimb unloading, decreased testicular and epididymal sperm counts, as well as testis weights, are observed (Kamiya et al., 2003; Usik & Ogneva, 2018). Prior sperm motility studies reported contradictory results (Engelmann et al., 1992; Ikeuchi et al., 2005). Clinical infertility diagnostics performed on cryopreserved human and bovine sperm on the ISS indicate that fertility‐related functions are altered inflight (Tash et al., 2019).

Relative to other organ systems, the gonads are highly sensitive to radiation exposure. Oxidative damage, double‐strand DNA breaks, oocyte and granulosa cell apoptosis (Mishra et al., 2016, 2017, 2018), and increased incidence of ovarian tumours (Salehi et al., 2008; Vanderhyden, 2005) are reportedly induced by IR exposure. High doses (3+ Gy) of IR increased the incidence of endometriosis in non‐human primates (Fanton & Golden, 1991; Wood et al., 1986). Though the testes are sensitive to IR, spermatogonial stem cells are relatively radioresistant compared with more differentiated stages (Alpen & Powers‐Risius, 1981; Sapp et al., 1990, 1992). Destruction of spermatogonia by IR involves similar mechanisms to the oocyte (Li, Zhang, Xie, et al., 2013; Li, Zhang, Miao, et al., 2013; Li et al., 2016; Zhao et al., 2016). Studies using mixed particle beam radiation on gonadal tissue, as well as on the uterus and other parts of the female reproductive tract, are needed.

The reproductive tissues are complex and versatile organs with important ties to many systems throughout the body and are known to be impacted by the spaceflight environment. Whether male or female, when the reproductive organs are not cycling normally on Earth, health problems can ensue, which might impact astronaut performance and resource utilization, should they occur in space. Expanding our understanding of the impact of the spaceflight environment on endocrine function, including hypothalamic–pituitary regulation, is critical if humans are to undertake multiyear missions beyond low Earth orbit.

## 12 GENITOURINARY

Urinary tract pathology develops frequently in the spaceflight environment and has been implicated in some of the most complex inflight medical cases to date. In 1985, one Shuttle mission was impacted when a case of prostatitis led to an episode of urosepsis, prompting the evacuation of the afflicted crewmember from the Mir space station. Urinary retention is a common occurrence among astronauts of both sexes, sometimes requiring urethral self‐catheterization to facilitate bladder emptying. There appears to be a microgravity‐unique mechanism to retention in this setting, further exacerbated by space motion sickness medication with anticholinergic side effects (Buckey, 2006). Anatomical differences between men and women presuppose an increased frequency of urinary tract infections (UTIs) among female astronauts, as seen terrestrially. While urinary retention occurs in crewmembers of both sexes, the incidence of UTIs appears to be higher in women than in men (Kumar et al., 2015).

Nephrolithiasis is one of the most concerning genitourinary risks in the spaceflight environment, largely due to the logistical challenges and mission impact associated with the medical management of the condition. The alterations in calcium metabolism seen in microgravity, in the setting of mild chronic dehydration, increase the risk of stone formation outright (N.A.). Terrestrially, approximately 75% of cases of nephrolithiasis occur among men. Ureteral occlusion by a stone and concomitant urinary congestion at the renal pelvis (termed hydronephrosis) may require prompt procedural attention in the form of lithotripsy, transurethral endoscopic stone retrieval or surgical excision. Of the five subtypes of renal stones, nearly 80% are calcium‐based, with a much higher burden of calcium oxalate‐containing stones (65–75%) as opposed to calcium phosphate (<5%). Struvite, or magnesium ammonium phosphate (triple phosphate), comprises approximately 15% of all urinary stones; these occur exclusively in individuals with recurrent UTIs, particularly those involving urease‐producing organisms and a urinary pH > 7.2. Struvite stones are the only subtype seen more often in women than in men. GCR appears to have an additive effect on the formation of kidney stones, in an oxidative stress‐mediated mechanism by which endothelial and mitochondrial dysfunction contribute to the diminished natriuresis seen among crewmembers (Pavlakou et al., 2018). Higher substrate concentration in the urine further increases the risk of crystal precipitation, leading to stone formation. Renal stones in male astronauts have been reported in the postflight setting in more than 30 instances (Sibonga, 2008). No known cases of nephrolithiasis have occurred among female astronauts, although postflight renal imaging in the absence of concerning symptoms may be inconsistent.

## 13 RADIATION

A review of the physiological effects of spaceflight would be incomplete without a dedicated discussion of the effects of IR, which is present in the spaceflight environment as GCR and is known to have deleterious effects with both acute and chronic exposure. Terrestrially, the effects of radiation‐induced genotoxicity are more apparent among tissues with rapid cellular turnover (i.e., gonads and skin); however, investigations following large‐scale exposures to radiation such as at Chernobyl, Hiroshima and Nagasaki revealed profound sequelae involving other parts of the body. It is important to note that GCR is only experienced in space; the IR‐induced damage encountered at these terrestrial sites is not necessarily indicative of the specific sequelae anticipated following long‐duration GCR exposure. There is still much to be learned regarding the effects of GCR and their relative doses, although collaborative work performed at ANSER by Dr Ron Turner and his team has yielded substantial progress in this area in recent years. As summarized in Table 4, the effects of IR exposure are vast (He et al., 2019; Mark, 2014; Morgan, 2003b; Narendran et al., 2019; Rubin, 1989; Tsai et al., 2005). Sex appears to be an important risk factor in predicting these outcomes, as radiation dose thresholds have historically been thought to be lower among women than men (Karagas et al., 1996; Lichter et al., 2000; Little, 2003; Yoshinaga et al., 2005). While the implications and sequelae of space radiation have been woven throughout this text, the critical nature of this subject warrants additional remarks.

**TABLE 4:** Ionizing radiation‐related physiological sequelae.

Organ/System	Effects	Explanation	Evidence source (terrestrial vs. space, animal vs. human, male ♂ vs. female ♀ vs. both ⚥ vs. not specified (NS))
Eye	Cataracts	Degradation of lens (Cucinotta, 2001).	Terrestrial and space, human, ⚥
Brain	Obstructs memory encoding Predisposes to encephalopathy	Neurotoxicity due to oxidative damage, dsDNA breakage and resultant cellular apoptosis (Asai & Kawamoto, 2008; Greene‐Schloesser et al., 2013; Hladik & Tapio, 2016).	Terrestrial, human and animal, ⚥/NS
Reproductive	Sterility (temporary or permanent) Birth defects Ovarian tumours	Increased susceptibility of gonadal tissue to oxidative damage with resulting dsDNA breakage and apoptosis (Rubin, 1989). Pre‐conception DNA damage in ovum and/or embryonic cellular damage (Lim et al., 2015).	Terrestrial, human and animal, ♀
Cardio‐pulmonary	Reduced exertional capacity OI Lung cancer	Cardiotoxicity at the level of the myocyte Fibrosis (proposed) and resultant compromise in exertional capacity (He et al., 2019). Genotoxicity and resultant oncogenic effect (Carey et al., 2007).	Terrestrial, human, NS
Endocrine	Thyroid cancer Breast cancer Reproductive organ dysfunction	Increased sensitivity of thyroid (Rubin et al., 1989) and breast tissue to IR (Morgan, 2003b). Increased sensitivity of gonadal tissue to oxidative damage (Morgan, 2003a; Rubin et al., 1989; Tsai et al., 2005).	Terrestrial and space, human and animal, ♀/NS
Blood	Global bone marrow suppression Haematological cancers	Genomic instability (Little, 2003).	Terrestrial and space, human, ⚥
Immune system	Generalized immunosuppression (due to global bone marrow suppression) Dysregulation of T‐cell response	Increased radiation sensitivity of cells involved in rapid turnover (Kufe, 2003).	Terrestrial and space, human and animal, ♀/⚥
Musculo‐skeletal	Increased bone resorption Reduced marrow stem cell population	Acute high dose (2+ Gy) and lower acute/continuous IR doses have differing results on bone loss (Alwood et al., 2017; Bandstra et al., 2009; Bokhari et al., 2019; Hamilton et al., 2006; Karim & Judex, 2014; Kondo et al., 2010; Willey et al., 2008; Wright et al., 2015; Yu et al., 2017).	Terrestrial, animal, ♂/♀
Genitourinary	Additive effect on formation of kidney stones	IR‐induced endothelial and mitochondrial dysfunction contribute to natriuresis (Pavlakou et al., 2018).	Terrestrial and space, human, ⚥
Skin	Skin cancers	IR‐induced oxidative damage with resulting DNA mutation (Karagas et al., 1996; Lichter et al., 2000; Yoshinaga et al., 2005).	Terrestrial, human, NS

Abbreviations: dsDNA, double‐stranded DNA; IR, ionizing radiation; OI, orthostatic intolerance.

NASA utilizes the “as low as reasonably achievable” (ALARA) principle for space‐permissible exposure limits or inflight radiation exposures (OCHMO 2023). In February 2021, NASA began to reconsider astronaut exposure limit to reflect a career exposure limit based upon a maximum 3% lifetime excess cancer mortality risk (600 mSV), as defined by the National Council on Radiation Protection, and an additional short‐term dose limit to prevent significant non‐cancer health effects inflight (250 mSV for SPEs), which are universal for all ages and sexes (OCHMO 2023). Individual astronaut risk of death due to radiation carcinogenesis are calculated by age and sex model parameters in the NASA cancer model (OCHMO 2023). Recent models estimate that astronauts on exploratory missions to Mars can expect to receive up to 2 mSv/day of GCR in transit, and up to 1 mSv/day on the Mars surface, separate from any solar particle events (SPEs), which carry higher, acute IR doses (Chancellor et al., 2018).

Over the last decade, this topic was discussed and evaluated by the National Academy of Medicine (NAM) Committee on Aerospace Medicine and the Medicine of Extreme Environments (Kahn et al., 2014). The committee was called upon to utilize the bioethical principles of *beneficence*, *nonmaleficence*, *autonomy* and *justice* to create safe, fair, and reliable guidelines for the conduct of future space missions. At the time of the study, the prevailing standards would have virtually eliminated women from exploration‐class missions to Mars: these standards were based predominantly on World War II‐era data in the form of an uncontrolled study from Nagasaki, Hiroshima and Chernobyl (Gilbert, 2001; Narendran et al., 2019), which overtly lacked any clear correlation with GCR or SPEs. The radiation limit was defined as any exposure which could stand to decrease total life expectancy due to cancer by 3%. Even now, this limit is misinterpreted by many researchers as implying that the increase in cancer mortality applies to the in‐mission phase. It must be emphasized that to date there is no clear evidence that women are at higher risk of radiation‐related effects *during the mission*, only that total lifespan might be reduced by 3% due to postflight cancer risk – among a population with a longer lifespan at baseline. It should be noted that this same constraining lifetime risk limit did not consider males who retired from the Astronaut Office and participated in particularly high‐risk off‐duty behaviour, such as the Reno Air Races, mountain climbing (e.g., Everest) and so forth. In calculating total acceptable radiation exposure limits (spaceflight, T‐38 sorties, dental X‐rays, etc.), female astronauts have been statistically more likely to reach this lifetime limit than males because they simply live longer – which has limited assignments to the ISS and may further limit exploration‐class mission involvement if not kept in check.

While the NAM report itself provides more extensive and granular detail, the application of its principles permits the following: (1) participation of both male and female crewmembers in a long‐duration mission in which NASA has determined that the benefits outweigh the risks, and that both male and female participation is obtained by means of individual informed consent, and (2) regardless of whether any woman (or man) has an increased risk of cancer later in life, due to family history or other genetic predispositions, should not prompt elimination, but rather thorough discussion and informed consent from the crewmember. Many NAM committee members expressed concern about facilitating an unintended paternalistic role in allowing the decision to be made to eliminate women from spaceflight altogether, simply because of the broad‐stroke assumption that women have a longer life expectancy than men and, consequently, an increased risk of developing cancer at some point in their lifetimes. In the interest of equality, every female crewmember should be afforded the opportunity to either accept or decline the risk of a 3% reduction in total lifetime expectancy due to the possibility of cancer. At the same time, in keeping with gold‐standard medical practice, any specific family history of cancer should be reviewed and discussed – with both male and female crewmembers – for the purpose of ensuring informed consent.

## 14 DISCUSSION AND RECOMMENDATIONS

Several major findings represent the most critical issues to be examined, to mitigate spaceflight‐associated medical risk and promote the success of missions to the Moon and Mars. It is imperative that the most salient sex‐linked differences observed in the terrestrial medical setting and/or in short‐duration missions to the ISS be studied more systematically in women during upcoming missions, including global immune dysregulation; increased levels of inflammatory markers, coagulation factors and leptin; correlation between body mass and the severity of ocular changes observed in SANS; increased incidence of OI, both on adaptation to microgravity and on reintroduction to the 1 *g* environment; increased severity of muscle atrophy and bone loss with prolonged unloading; differences in the incidence and type of UTIs; and susceptibility to certain cancers after exposure to IR. We strongly recommend that radiation dose limits be recalibrated, considering the context provided above. Given the recent concern of a global hypercoagulable state triggered by the spaceflight environment, special attention should be given to a better understanding of the multifactorial aetiology and possible sex‐linked influences. This will inform the needed development of novel strategies to mitigate clot risk and plan precision interventions.

The durability of crew health and wellness on extended‐duration missions will depend on the space medicine community leveraging advanced innovative approaches to prevent, detect and address the myriad health problems that arise inflight. As spacesuit design evolves, an ergonomic fit for both male and female frames should be prioritized, to optimize crew ergonomics and performance on extravehicular activities. To improve current astronaut health as well as develop safe strategies for longer missions, a paradigm shift is needed, which would surpass the current response planning for medical conditions and include an emphasis on early detection, precision interventions, and closed‐loop decision support for personal health and medical management. This proactive and individualized approach would likely benefit both female and male astronauts. This will entail the deployment of integrated components not currently available on the ISS, including integrated sensing technologies, advanced computational capabilities with edge analytics and role‐specific feedback mechanisms. Several well‐validated and minimally intrusive monitoring devices are available which record physiological metrics, biometrics, sleep, activity patterns, and even facial expression and body language in interpersonal interactions. Maximizing the use of such technologies may provide meaningful insights into the physical and psychological states of crew members and provide insights into crew dynamics. Psychological parameters and crew cohesion historically have been much more difficult to measure in an unobtrusive manner. Awareness of individual CR may allow more tailored treatment of CR dysregulation and sleep disturbance with non‐pharmacological interventions such as wavelength‐specific light therapy, exercise, meditation and sound recordings and perhaps reduce the need for sleep medications. These examples of technology applications may be deployed strategically to gain more detailed knowledge of individual responses to spaceflight and to minimize health risks, including those that affect the sexes differently.

## 15 CONCLUSION

Candidates are screened rigorously prior to selection to the astronaut corps, to identify medical conditions on the ground that might predispose them to problems inflight. However, even the healthiest of individuals run the risk of developing a number of diseases *de novo* when exposed to the spaceflight environment (Crucian et al., 2016). Many health risks in space appear to impact women differently from men, rendering thoughtful medical preparedness for long‐duration spaceflight a particularly complex but necessary undertaking. To optimize the health of all crewmembers, it is imperative that more individual‐level data be obtained and leveraged, and that more female astronauts be selected for operational roles on upcoming missions. When more women fly, more data will be available for further identification of sex‐based health risks and the implementation of precision interventions, in spaceflight and on Earth.

## AUTHOR CONTRIBUTIONS

Idea and conceptualization: Danielle J. Carroll and Aenor J. Sawyer. Literature search: Millie Hughes‐Fulford, Danielle J. Carroll, and Heather C. M. Allaway. Manuscript draft: Millie Hughes‐Fulford, Danielle J. Carroll, and Heather C. M. Allaway. Manuscript review and editing: Millie Hughes‐Fulford, Danielle J. Carroll, Heather C. M. Allaway, Bonnie J. Dunbar, and Aenor J. Sawyer. All authors have read and approved the final version of this manuscript and agree to be accountable for all aspects of the work in ensuring that questions related to the accuracy or integrity of any part of the work are appropriately investigated and resolved. All persons designated as authors qualify for authorship, and all those who qualify for authorship are listed. Millie Hughes‐Fulford passed away prior to the completion of the final manuscript review. Danielle J. Carroll began this project while at the University of California San Francisco and is currently at the University of Colorado Boulder. Heather C. M. Allaway began this project while at Texas A&M University and is currently at Louisiana State University.

## CONFLICT OF INTEREST

The authors declare no conflicts of interest.
